# Outbreak of highly pathogenic avian influenza in Ghana, 2015: degree of losses and outcomes of time-course outbreak management

**DOI:** 10.1017/S095026882000045X

**Published:** 2020-02-17

**Authors:** W. Tasiame, S. Johnson, V. Burimuah, E. Akyereko, P. El-Duah, E. Amemor, B. O. Emikpe, E. W. Owiredu

**Affiliations:** 1 School of Veterinary Medicine, Kwame Nkrumah University of Science and Technology, Kumasi, PMB, UPO, Kumasi 00233, Ghana; 2 School of Veterinary Medicine, CBAS, University of Ghana, Legon, Accra, Ghana; 3 Disease Surveillance Department, Ghana Health Service, Accra, Ghana; 4 Kumasi Centre for Collaborative Research in Tropical Medicine, PMB, UPO, Kumasi 00233, Ghana; 5 Department of Molecular Medicine, School of Medicine and Dentistry, Kwame Nkrumah University of Science and Technology, PMB, UPO, Kumasi 00233, Ghana

**Keywords:** HPAI, management, outbreak, time-course

## Abstract

This retrospective study highlights the degree of losses and time-course through which the 2015 highly pathogenic avian influenza (HPAI) outbreaks in Ghana were managed. A total of 102 760 birds from 35 farms across five regions in Ghana included in this study were affected. Out of this, 89.3% was from the Greater Accra region. Majority of the birds were culled (94.2%). Adult layers were most affected and destroyed (64.0%), followed by broilers (13.7%). Event initiation to reporting averaged 7.7 ± 1.3 days (range: 1–30 days). Laboratory confirmation to depopulation of birds averaged 2.2 ± 0.5 (0–15) days while depopulation to disinfection took 2.2 ± 0.7 (0–20) days. Overall, some farms took as long as 30 days to report the outbreak to the authorities, 15 days from confirmation to depopulation and 20 days from depopulation to disinfection. On average, outbreak management lasted 12.3 (2–43) days from event initiation to depopulation. The study reveals a significant number of avian losses and delays in HPAI reporting and management by the authorities in Ghana during the 2015 outbreak. This poses a high risk of spread to other farms and a threat to public health. Awareness creation for poultry farmers is necessary for early reporting, while further study is required to set thresholds for the management of such outbreaks by veterinary departments.

## Introduction

Avian influenza (AI) is an epidemic viral infection caused by avian influenza Type A viruses [1]. There are two distinct types of influenza A viruses that infect poultry based on their disease-causing capabilities. Whereas the highly pathogenic avian influenza (HPAI) causes severe illness in domestic birds with high mortalities, the low pathogenic avian influenza (LPAI) manifests milder symptoms [2]. Direct contact with infected and dead birds is probably the most reported mode of transmission to humans [3]. Apart from its public health significance, AI exerts an enormous toll on the socio-economic status of countries during outbreaks [4].

Historically, the major spread of HPAI H5N1 virus began in eastern and southeastern Asia from 2003 through 2004. In 2005–2006, HPAI had moved westward across Asia into Europe, the Middle East and Africa [5]. The AI epidemic in Asia resulted in the infection of all species of domestic poultry within 8 years (1997–2005) [6]. In Africa, HPAI was first reported in Nigeria in 2006 [7]. This was also the first reported outbreak of the H5N1 Asian strain on the African continent. Egypt was the second African country to be infected with H5N1. Over 30 million birds were reportedly culled; an estimated 250 000 jobs and a high number of human lives were lost as a result [8]. The Food and Agriculture Organization (FAO) of the United Nations (UN) also reported HPAI H5N1 outbreaks in Togo and 10 other African countries from February 2006 to July 2008 [9].

In Sub-Saharan Africa, countries endemic with HPAI H5N1 include Burkina Faso, Cameroon, Côte d'Ivoire, Niger, Nigeria, Togo and Ghana [10]. It is believed that the outbreaks of AI in neighbouring countries may have influenced the outbreaks in Ghana. After the first outbreak of HPAI and its management in Ghana, several programmes were carried out to educate the public on biosecurity measures. Active AI surveillance was conducted on different types of birds using real-time polymerase chain reaction (PCR) with no positive case for influenza A [8]. Ghana did not report any further outbreaks of HPAI since the 2006–2007 wave until Nigeria recommenced reporting positive cases in January 2015 [11]. The primary administrative regions in Ghana affected were Greater Accra, Ashanti, Central, Eastern, Volta and Western region, and a total of 63 outbreaks have been reported as of November 2016 [10]. Though the provenance of HPAI H5N1 in Ghana has not been explicitly outlined, a report by Mabbett indicates a 98.8–99.6% homology with isolates from Burkina Faso, Cote d'Ivoire, Nigeria and Sudan [12].

The detrimental effects of HPAI on the poultry industry as well as its threat to public health necessitated the establishment of Technical Committee of Experts on Prevention and Control of Avian Influenza tasked with developing strategies for the prevention of the disease, surveillance networks and emergency preparedness plans for disease containment [13, 14]. Nonetheless, insufficient financial and logistical resources, weak Veterinary Services, lax border controls on animal movements, conflicts and inappropriate governance provide an enabling environment for the spread of HPAI and other transboundary animal diseases [15]. This is evidenced in the recent confirmation of AI virus subtype H9N2 in 2018 [16].

The poultry industry in Ghana remains at its juvenile stage. There is thus a critical need to monitor all health-related circumstances that endanger it. HPAI not only infects poultry but also the humans who tend for the birds [17]. It is therefore imperative to have sufficient knowledge on the outbreaks of AI that occurred in Ghana and the time-course through which these outbreaks were managed. This information would equip policymakers with the necessary information to develop apt strategies to improve upon the management of future outbreaks. It is against this background that this study analysed the 2015 AI outbreak data in Ghana to evaluate the degree of losses and time-course of its management.

## Methods

### Study area and outbreak data

**Fig. 1.**
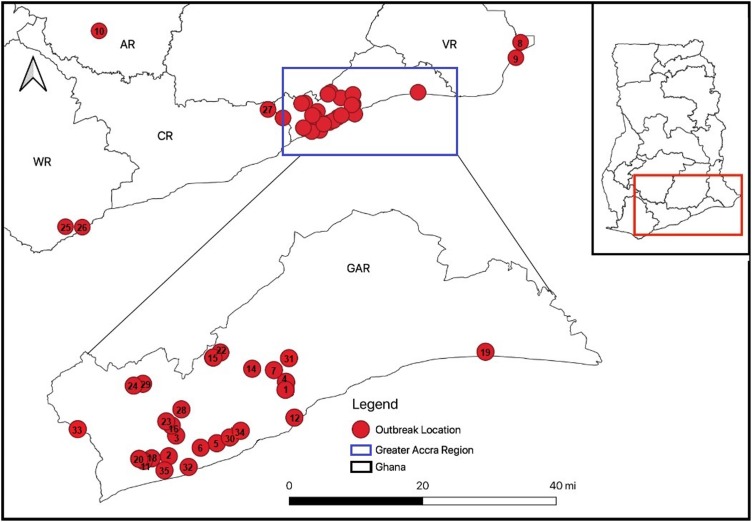
Map showing the 2015 HPAI outbreaks sites and the time of outbreaks. The numbers represent the time-lines for each outbreak (numbers do not add up because some outbreaks were too close and thus overlapped).

The study was a retrospective analysis of AI outbreak data from Ghana in 2015. Ghana is located along the Gulf of Guinea and the Atlantic Ocean, in the sub-region of West Africa. Ghana spans a landmass of 238 535 km^2^, and it is bordered by Ivory Coast to the west, Burkina Faso to the north, Togo to the east and the Gulf of Guinea and the Atlantic Ocean to the south. It lies on the latitude 7.9528 and the longitude −1.0307 [18]. Study sites included were the Ashanti, Central, Volta, Western and Greater Accra regions, which constitute five of the total 10 administrative regions of Ghana. A total of 35 farms across the five regions were included in the study. Areas captured were those who reported AI outbreaks within the study period. All outbreak sites and the time-line for each outbreak were geo-referenced (Fig. 1).

Data for this study were obtained from the epidemiology unit of the Veterinary Services Department under the Ministry of Food and Agriculture, Ghana. Checks were made from the affected region and district veterinary offices for confirmation before use for this review. The retrieved data consist of AI outbreaks from January through December of 2015. Data captured included AI cases from the backyard, small-scale commercial and free-range poultry. Other variables assessed included bird population, type of birds, number of birds destroyed, natural deaths (died prior to the depopulation events), and dates of initiation of event, confirmation, depopulation and disinfection. Districts and regions affected in these outbreaks were also captured. Avian species covered included chickens, ducks, pigeons, turkeys and guinea fowls.

Approval for this study was obtained from the National Epidemiology Unit of Veterinary Services of Ghana, the source that generated these data.

### Statistical analysis

Statistical analysis was performed using the R Language for Statistical Computing version 3.6.0 [19]. Categorical and continuous data were expressed as frequencies (percentages) and means ± standard error of the mean, respectively. Independent *t* test and one-way ANOVA were used to determine the significance of differences between farm sizes, farm types and administrative zones in relation to time-course management, respectively. A *P*-value <0.05 was considered statistically significant.

## Results

Overall, 102 760 birds originating from 35 farms across five major regions were included in the study. Out of this figure, the Greater Accra region recorded the highest proportion (89.3% (91771/102760)) and the remainder was distributed among the other four regions. A total of 5976/102760 (25.3%) of the birds affected died naturally as a result of HPAI (prior to the depopulation events), with the Volta region recording the highest number of natural losses (2450/3400 (72.1%)). On the other hand, the Ashanti region recorded the highest proportion of avian losses by culling (1883/96784 (96.7%)). Approximately 98% (1628/1664) of the eggs destroyed originated from Greater Accra (Table 1).

**Table 1.** Distribution of avian losses by avian influenza affected regions in Ghana, 2015

Region	No of farms affected	Bird population	Bird destroyed	Natural deaths	Eggs destroyed (crates)
Ashanti	1	1948 (1.9)	1883 (96.7)	65 (3.3)	12 (0.7)
Central	1	510 (0.5)	476 (93.3)	34 (6.7)	4 (0.2)
Volta	2	3400 (3.3)	950 (27.9)	2450 (72.1)	18 (1.1)
Western	2	5131 (5.0)	3459 (67.4)	1672 (32.6)	2 (0.1)
Greater Accra	29	91 771 (89.3)	70 016 (76.3)	21 755 (23.7)	1628 (97.8)
Total	35	102 760 (100)	96 784 (94.2)	25 976 (25.3)	1664 (100)

Percentages for bird population and number of eggs destroyed were calculated by column. Percentages for birds destroyed and natural deaths were by rows, using bird population as the denominator.

**Fig. 2.**
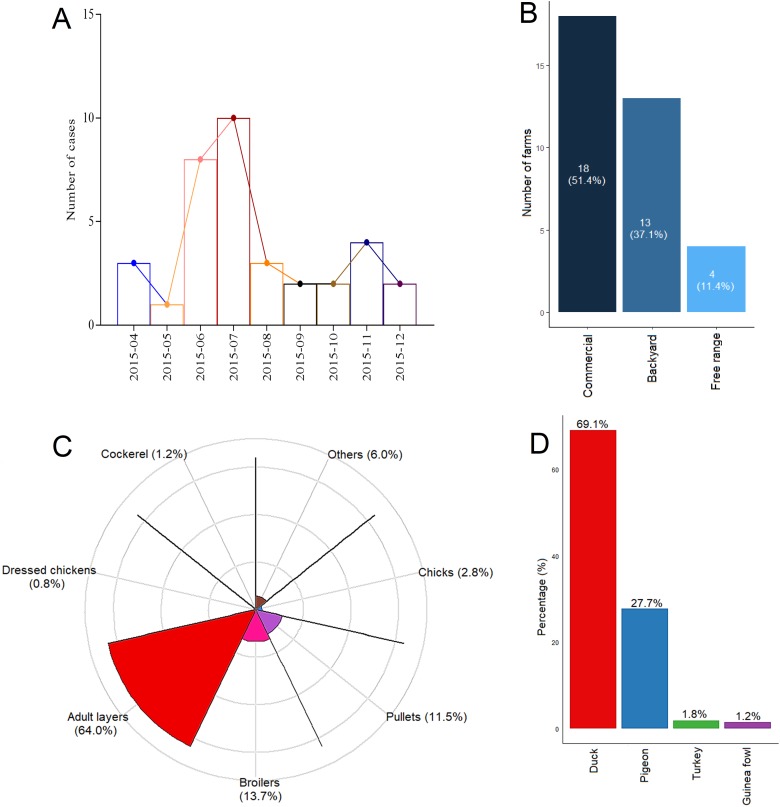
Epicurve of outbreaks and descriptive statistics of the type of farm and birds affected. (a) Epicurve showing the number of outbreaks per month. (b) Distribution of the type of farm affected. (c) Avian influenza losses by the type of bird (percentages were calculated using the total number of birds destroyed). (d) Avian influenza losses for birds other than domestic fowl (percentages were calculated using the total number of birds destroyed other than domestic fowl).

The outbreak began in April 2015 with three incidents that continued spreading throughout the year. Cases peaked in June through July before recording a downward trend in subsequent months (Fig. 2a). Most of the farms affected were small-scale commercial farms (51.4% (18/35)) (Fig. 2b) and adult layers were the most affected and destroyed (61942/96784 (64.0%)). Available dressed chickens ready for market were also destroyed (775/96784 (0.8%)) (Fig. 2c). Additionally, out of the 6.0% losses for birds other than domestic fowls, ducks recorded the greatest loss (4012/5807 (69.1%)) (Fig. 2d).

**Fig. 3.**
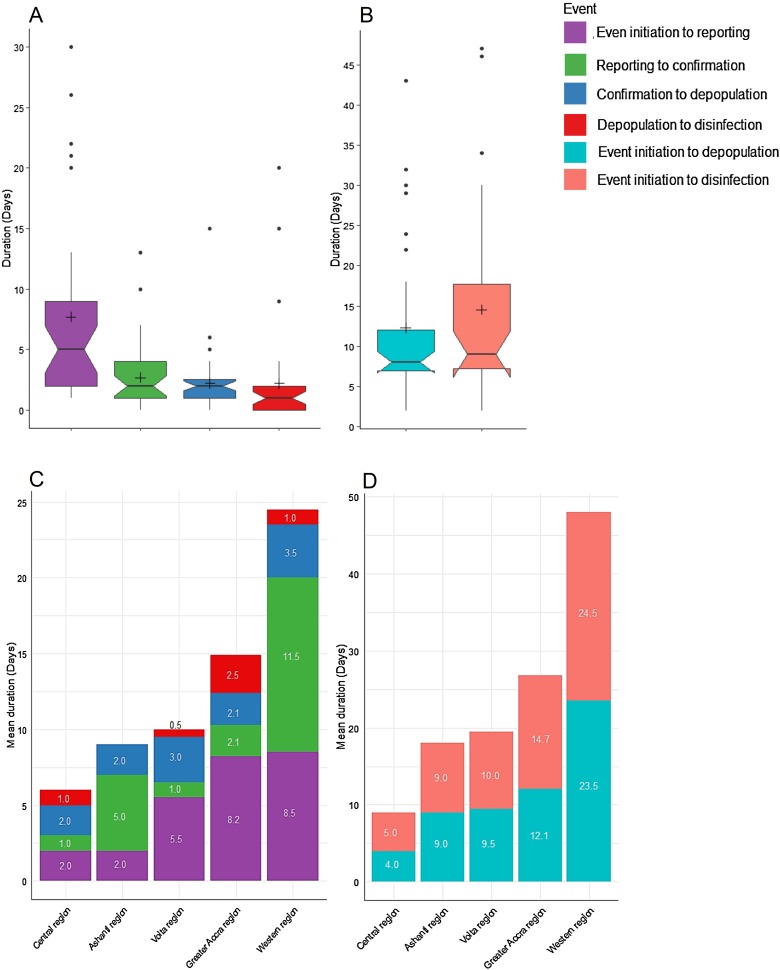
Time-course management of HPAI outbreak. (a, b) Time-course from event initiation to disinfection across all outbreak sites. (c, d) Time-course from event initiation to disinfection stratified by administrative zones.

The average duration from the initiation of the event to reporting was 7.7 ± 1.3 (range: 1–30) days while that from reporting to laboratory confirmation was 2.7 ± 0.5 (0–13) days. The duration from laboratory confirmation to depopulation of birds was 2.2 ± 0.5 (0–15) days and depopulation to disinfection lasted for 2.2 ± 0.7 (0–20) days. Overall, it took as long as 30 days to report the outbreak after initiation of the event for some farms, 15 days from confirmation to depopulation and 20 days from depopulation to disinfection. Additionally, for some cases, it took 13 days for laboratory confirmation after reporting (Fig. 3a). Taken together, the average duration from event initiation to depopulation was 12.3 ± 1.7 (2–43) days whereas from event initiation to disinfection was 14.6 ± 2.0 (2–47) days (Fig. 3b). Strikingly, upon stratification by administrative zones, Western region, despite presenting with only two outbreak cases, recorded the highest mean duration from event initiation to depopulation, although not statistically significant (*P*-value >0.05 for all comparison) (Fig. 3c and 3d, Supplementary Table S1).

**Fig. 4.**
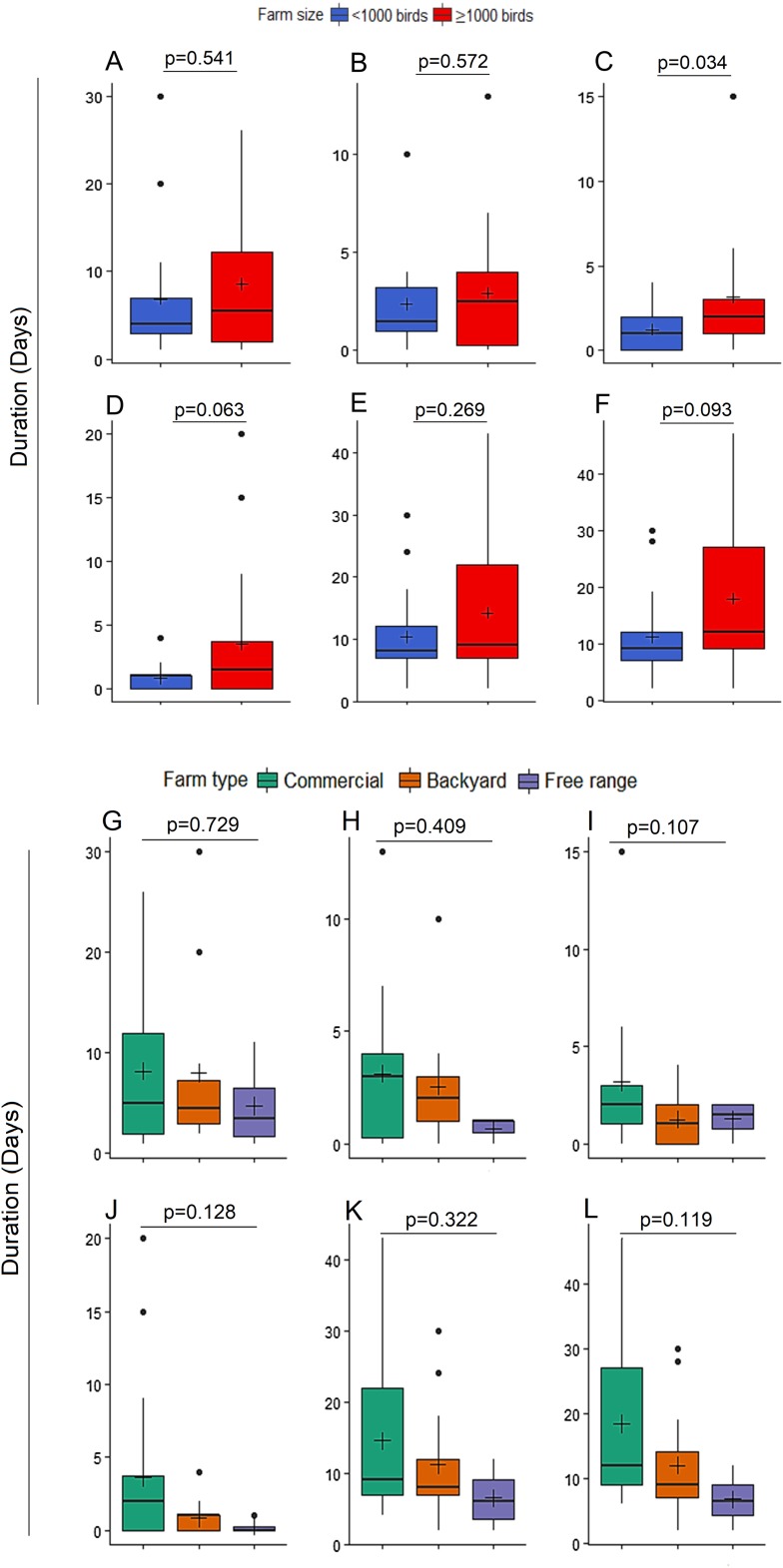
Time-course management of HPAI outbreak by farm size and farm type. (a, g) Event initiation to reporting. (b, h) Reporting to confirmation. (c, i) Confirmation to depopulation. (d, j) Depopulation to disinfection. (e, k) Event initiation to depopulation. (f, l) Event initiation to disinfection.

To evaluate the influence of farm size – defined as the number of poultry on each farm – and farm type on the time-course of the management, we grouped farms into those having <1000 and ≥1000 birds and commercial, backyard and free-range, respectively. Farms with ≥1000 birds consistently presented with higher duration for management across all events; however, only the time-course from confirmation to depopulation was statistically significant (1.2 ± 0.3 *vs.* 3.2 ± 0.8 days, *P* = 0.034). No statistically significant differences were observed for farm type (Fig. 4).

## Discussion

In 2015, Ghana experienced HPAI outbreaks which began in April and ended in December, with peak outbreaks in June–July. The outbreak has been proposed to have originated from Nigeria. Sequence analysis of the viral hemagglutinin revealed that the 2015 Ghana outbreak strain possessed a multi-basic cleavage site (RERRRKR/GLF), as common to HPAI H5N1 viruses [20]. Phylogenetic analysis confirmed that the strain belonged to clade 2.3.2.1c. Genetic analysis of polymerase basic protein 2 showed that the strain lacked the known human adaptive signatures E627K or D701N [11]. Nucleoprotein sequences of isolates from Ghana revealed 99% homology with the Nigeria 2015 outbreak strain, confirming the high probability of the origin of the virus [11]. Small-scale commercial farms were the most affected and a total of 102 760 bird losses were incurred. The losses were predominantly in the Greater Accra region. This was expected because 29 out of the 35 farms affected were located in the region. The region is also home to the international airport and main seaport which could have facilitated free and increased the inflow of persons and goods, enabling increased chance of spreading. Undoubtedly, the increased number of affected birds in the Greater Accra region may also be attributed to the proximity of farms within the region. Congruently, Saidu *et al*. [1] observed that the proximity of farms during outbreaks facilitated rapid spreading. Turkson also reported that the free flow of goods, people, poultry and poultry products, through approved and unapproved borders, is a major means of introducing diseases such as HPAI into Ghana [21]. Furthermore, trading of live birds once a possible outbreak is announced may be partly involved. Morris *et al*. [22] indicated that, in countries where disease outbreaks are poorly controlled, farmers usually respond to the situation by selling their birds to reduce their financial losses. This attitude may have also facilitated the spread of the disease from the source of the outbreak in Accra, within the region and to neighbouring regions such as the Volta region which shares a border with Greater Accra, with several entry points for the movement of good and people. Outbreaks in other regions are likely to be due to the poorly-regulated trans-regional trade of live birds, a fairly common practice by poultry farmers in the country.

The predominant species of birds affected were domestic chickens, followed by ducks, pigeons, turkeys and guinea fowls. Among the chickens, adult layers were the most affected and destroyed. This finding is in harmony with the findings of Saidu *et al*. [1] and Akanbi *et al*. [23] in Nigeria who reported a similar trend. The FAO indicated that domestic fowl, ducks, geese, turkeys, guinea fowl, quail and pheasants are all susceptible to AI, although outbreaks predominantly occur in domestic fowl and turkeys [24]. Other studies have also reported that other avian species such as ducks, turkeys, geese, etc., play vital roles in the epidemiology of AI [23, 25, 26]. Moreover, ducks have been implicated in the transmission of LPAI and HPAI virus among themselves and other domestic birds via direct or indirect contacts [27]. Thus, small-scale poultry farms that rear birds other than domestic fowls also contribute to the transmission of AI. It should, however, be noted that since Newcastle disease was not assessed, the significance of pigeons in this study may be limited.

The strength of this study is in reporting the time-course of management during the 2015 AI outbreak in the country. The first case of HPAI H5N1 among poultry in Ghana was reported in 2007 [11, 28]. The outbreak occurred in three regions: Greater Accra, Volta and Brong Ahafo. After containing the 2007 HPAI H5N1 outbreak, active AI surveillance was initiated. Due to the high losses during the 2007 outbreak, it was expected that the influenza surveillance will consider the limitations and build on the past experiences for a more resilient and timely approach towards new outbreaks. However, we observed considerable delays from the onset of clinical signs through the confirmation of disease to the initiation of control measures during the 2015 HPAI H5N1 outbreak. Timely reporting and confirmation are key to control the spread of the disease. Delays in these events affect downstream measures such as the times for destruction of all birds on affected farms, disinfection of affected farms and restricted movement of poultry and poultry products. Of note, there were also delays from confirmation to depopulation and subsequent disinfection which may have aggravated the rapid dissemination of the disease across regional borders. In 2004, Japan reported HPAI H5N1 cases in four different premises [29]. Out of this, three farms reported the cases to the appropriate authorities within a day of onset of clinical signs and the disease was successfully eradicated within three-and-a-half months, following an eradication campaign. This underscores the significance of early reporting and confirmation in the control of AI and could be the major reason why the outbreak in Ghana took over 9 months for complete containment to be achieved. We attribute the delay in reporting partly to a lack of knowledge of the disease among farmers. Education of farmers regarding AI, its pathology, control and possible preventive measures will be thus indispensable in the stride against AI in Ghana and Africa.

In Ghana, before the transportation of suspected HPAI samples to the International Reference Laboratory for Newcastle Disease and Avian Influenza, Italy, for confirmation, the samples undergo initial internal screening. The initial diagnosis is done through rapid tests, conventional PCR and currently, real-time PCR. The laboratory is however located only in Accra, the capital of Ghana. Thus, distance, coupled with unfavourable transportation routes, may have also played a role in the delays observed in this study. Inadequate supply of consumables such as laboratory reagents and bureaucratic delays has also been identified as a major limiting factor to the rapid containment of the HPAI in Ghana and Africa. Internal restructuring, personnel training and continuous supply of diagnostic resources could help to rapidly and effectively contain future outbreaks.

We also observed delays in depopulation and disinfection after confirmation which could have facilitated the spread of the disease because AI virus can persist in distilled water or sterilised environmental water for several days to months [30–33]. Therefore, to avert escalation and spread, it is of great importance to quickly depopulate infected birds following an immediate diagnosis on each affected farm.

The farms affected during the 2015 HPAI outbreak consisted of varying flock population. A study by Loth *et al*. in Indonesia [34] and Tiensin *et al*. in Thailand [35] reported an association between farm size, type and HPAI. The influence of geographic location has also been reported by Ssematimba *et al*. [36]. In this study, we also sought to examine the influence of farm size, type and geo-location on the temporal variations for the control of the HPAI outbreak. Interestingly, higher delays in management were observed consistently in both larger farms and small-scale commercial farms. Additionally, although the Western region had only a few outbreak cases, the region recorded the most delays across most containment measures. All the farms in the Western region were free-range with relatively small flock size. Evidence suggests that whereas larger-scale farmers are more likely to observe suspected cases, report to the appropriate authorities and request for early feedback, encouraged by the compensation that they received, small-scale farmers are not inclined to report their few dead poultry [35]. Consequently, the outbreaks in small-scale farms such as those in the Western region may have been overlooked. This highlights the need for heightened vigilance and strengthened surveillance even on smaller farms.

Low biosecurity measures observed on Ghanaian farms [21, 37], coupled with delays in containment strategies, increase the likelihood of spread of the virus and a possible toll of AI on the poultry industry if appropriate measures are not timely enforced.

## Conclusion

The study reports a significant number of avian losses during the 2015 HPAI outbreak in Ghana. There were delays in reporting the HPAI outbreaks to the veterinary authorities in Ghana and from confirmation to depopulation and disinfection. These delays pose a high risk for spread to other farms and human population. Awareness creation for poultry farmers is necessary for early reporting, whereas further studies are required to set thresholds for the management of such outbreaks by veterinary personnel. Furthermore, continuous active surveillance of the disease is imperative to quickly identify cases before outbreaks become aggravated.

## Supplementary material

For supplementary material accompanying this paper visit https://doi.org/10.1017/S095026882000045X. click here to view supplementary material
