# Supplementary material for: Outbreak of highly pathogenic avian influenza in Ghana, 2015: degree of losses and outcomes of time-course outbreak management
Source: Epidemiol Infect. 2020 Feb 17;148:e45. doi: 10.1017/S095026882000045X (PMC7058832; doi:10.1017/S095026882000045X)
Supplement: Supplementary file 1 [file S095026882000045Xsup001.docx]

***Epidemiology and Infection***

**Outbreak of Highly Pathogenic Avian Influenza in Ghana, 2015: outcomes of time-course outbreak management**

W. Tasiame, S. Johnson, V. Burimuah, E. Akyereko, P. El-Duah, E. Amemor^1^, B. O. Emikpe, E. W. Owiredu

**Supplementary Material**

**Supplementary Table S1. Comparison of time-course management by administrative zones**

| **Event/Region** | **Greater Accra** | **Volta** | **Central** | **Western** | **Ashanti** | **p-value** |
| --- | --- | --- | --- | --- | --- | --- |
| Event initiation to reporting | 8.2±1.6 | 5.5±3.5 | 2.0±0.0 | 8.5±4.5 | 2.0±0.0 | 0.338 |
| Reporting to confirmation | 2.1±0.3 | 1.0±1.0 | 1.0±0.0 | 11.5±1.5 | 5.0±0.0 | 0.420 |
| Confirmation to depopulation | 2.1±0.5 | 3.0±3.0 | 2.0±0.0 | 3.5±0.5 | 2.0±0.0 | 0.741 |
| Depopulation to disinfection | 2.5±0.9 | 0.5±0.5 | 1.0±0.0 | 1.0±0.0 | 0.0±0.0 | 0.388 |
| Event initiation to depopulation | 12.1±2.0 | 9.5±1.5 | 4.0±0.0 | 23.5±5.5 | 9.0±0.0 | 0.983 |
| Event initiation to disinfection | 14.7±2.3 | 10.0±2.0 | 5.0±0.0 | 24.5±5.5 | 9.0±0.0 | 0.756 |
